# Independent and Distinct Associations of FABP4 and FABP5 With Metabolic Parameters in Type 2 Diabetes Mellitus

**DOI:** 10.3389/fendo.2020.575557

**Published:** 2020-09-23

**Authors:** Masato Furuhashi, Ichiro Sakuma, Takeshi Morimoto, Yukimura Higashiura, Akiko Sakai, Megumi Matsumoto, Mio Sakuma, Michio Shimabukuro, Takashi Nomiyama, Osamu Arasaki, Koichi Node, Shinichiro Ueda

**Affiliations:** ^1^Department of Cardiovascular, Renal and Metabolic Medicine, Sapporo Medical University School of Medicine, Sapporo, Japan; ^2^Caress Sapporo Hokko Memorial Clinic, Sapporo, Japan; ^3^Department of Clinical Epidemiology, Hyogo College of Medicine, Nishinomiya, Japan; ^4^Department of Diabetes, Endocrinology and Metabolism, Fukushima Medical University, Fukushima, Japan; ^5^Department of Diabetes, Metabolism and Endocrinology, International University of Health and Welfare Ichikawa Hospital, Ichikawa, Japan; ^6^Department of Cardiology, Tomishiro Central Hospital, Tomigusuku, Japan; ^7^Department of Cardiovascular Medicine, Saga University, Saga, Japan; ^8^Department of Pharmacology and Therapeutics, University of the Ryukyus, Okinawa, Japan

**Keywords:** adipokine, fatty acid-binding protein, dipeptidyl peptidase-4 inhibitor, stain, diabetes mellitus

## Abstract

**Objective:** Among fatty acid-binding proteins (FABPs), secreted forms of FABP4 and FABP5, which are expressed in adipocytes and macrophages, act as bioactive molecules. We investigated concentrations of FABP4 and FABP5 in patients with type 2 diabetes mellitus.

**Methods:** As a sub-analysis study of the Randomized Evaluation of Anagliptin vs. Sitagliptin On low-density lipoproteiN cholesterol in diabetes (REASON) trial, 256 patients (male/female: 146/110, age: 68 ± 10 years) with type 2 diabetes mellitus and dyslipidemia who were receiving statin therapy were recruited. Patients who had been treated with a thiazolidinedione were excluded.

**Results:** Several drugs which may modulate FABP4 levels including statins, dipeptidyl peptidase-4 inhibitors and angiotensin II receptor blockers had been administered in 100, 81, and 51% of the recruited patients, respectively. The level of FABP4, but not that of FABP5, was significantly higher in females than in males. Multivariable linear regression analysis demonstrated that waist circumference (β = 0.21), estimated glomerular filtration rate (β = −0.31), triglycerides (β = 0.16), and FABP5 (β = 0.39) were independent predictors of FABP4 level after adjusting age and sex. On the other hand, FABP5 level was independently associated with levels of FABP4 (β = 0.57) and high-density lipoprotein (HDL) cholesterol (β = −0.12).

**Conclusions:** Concentrations of FABP4 and FABP5 are independent predictors of each other in patients with type 2 diabetes mellitus. There are distinct independent associations of FABP4 with renal dysfunction, adiposity and hypertriglyceridemia and there is a distinct independent association of FABP5 with a low HDL cholesterol level in type 2 diabetic patients with dyslipidemia at high risks for cardiovascular disease who are receiving statin therapy.

## Introduction

Fatty acid-binding proteins (FABPs) as lipid chaperones are about 14–15-kDa proteins which can bind long-chain fatty acids and facilitate lipid transport to several organelles in cells (1, 2). Among FABPs, FABP4, also known as aP2 or adipocyte FABP, is mainly expressed in adipocytes and macrophages and is related to the development of metabolic disorders including insulin resistance and atherosclerosis (3–5). Small molecule specific FABP4 inhibitors and/or FABP4 neutralizing antibodies can be novel therapeutic strategies against metabolic disorders and vascular remodeling (6–8). Another FABP, FABP5, also referred to as mal1 or epidermal FABP, is expressed in several types of cells including skin, macrophages and adipocytes (1). Experimental studies using FABP5-knockout mice showed the relationship of FABP5 with insulin resistance and atherosclerosis (9, 10).

Lipolysis-associated secretion of FABP4 from adipocytes is caused by a non-classical secretion pathway (7, 11) despite the lack of signal peptides (1), and secreted FABP4 has effects as an adipokine on insulin resistance, atherosclerosis and vascular remodeling (7, 8, 12). An elevation of FABP4 concentration is associated with metabolic disorders and cardiovascular events (13–23). It has recently been shown that FABP4 concentration is modulated by several therapeutic drugs for diabetes mellitus, dyslipidemia and hypertension (24–30). Secretion of FABP5 from cells is also confirmed (31), but the mechanism of secretion is still unclear. It has been demonstrated that exogenously treated FABP4 and FABP5 distinctly act on metabolic and transcriptional response in adipose-derived stem cells that are present near adipocytes (31). It has been shown that circulating FABP5 level is related to cholesterol efflux capacity as a quality marker of high-density lipoprotein (HDL) cholesterol and atherosclerosis in coronary and carotid arteries (14, 32–34).

However, little is known about the association of concentrations of FABP4 and FABP5 with metabolic markers in a real-world clinical setting. We investigated concentrations of FABP4 and FABP5 in patients with type 2 diabetes mellitus who were receiving statin therapy at high risks for cardiovascular disease.

## Methods

### Study Patients

Study patients were recruited from the Randomized Evaluation of Anagliptin vs. Sitagliptin On low-density lipoprotein cholesterol in diabetes (REASON) trial (35, 36). In the REASON trial, 52-weeks treatment with anagliptin, a dipeptidyl peptidase-4 (DPP-4) inhibitor, was shown to be associated with a greater reduction in level of low-density lipoprotein (LDL) cholesterol than was treatment with sitagliptin, another DPP-4 inhibitor, in patients with type 2 diabetes mellitus and with LDL cholesterol levels of >100 mg/dL who were receiving statin therapy (36). The REASON trial was registered (Clinicaltrials.gov number, NCT02330406) and was conducted in accordance with the Ethical Guidelines for Medical and Health Research Involving Human Subjects in Japan and the Declaration of Helsinki. Institutional review boards at the University of the Ryukyus and each center approved the protocol. Sub-analysis studies using stored serum samples were planned in the protocol and were conducted by the decision of central committee. Written informed consent including sub-analysis studies was obtained from all enrolled patients. In the present study, which was one of the sub-analysis studies, circulating levels of FABP4 and FABP5 were investigated. Since FABP4 is a target of peroxisome proliferator-activated receptor γ (PPARγ) (37, 38), patients who had been treated with a thiazolidinedione, which is a PPARγ agonist, were excluded. After exclusion, a total of 256 patients (male/female: 146/110, age: 68 ± 10 years) were included in the present study.

### Measurements

Clinical characteristics, including age, sex, waist circumference, body mass index (BMI) calculated as body weight (kg) divided by height (m) squared, habits of alcohol drinking and smoking and use of concomitant drugs were evaluated. Fasting glucose, creatinine, blood urea nitrogen, γ-glutamyl transpeptidase (γGTP), alanine aminotransferase (ALT), and aspartate transaminase (AST) were measured in each participating center. Estimated glomerular filtration rate (eGFR) was calculated using a formula for Japanese people as previously reported (39). Hemoglobin A1c (HbA1c) by the National Glycohemoglobin Standardization Program (NGSP), LDL cholesterol by direct measurement, triglycerides, HDL cholesterol, total cholesterol and insulin were measured at a core laboratory (SRL, Tokyo, Japan). Non-HDL cholesterol was calculated as total cholesterol minus HDL cholesterol. Homeostasis model assessment of insulin resistance (HOMA-R), an indicator of insulin resistance, was calculated by the previously reported formula: fasting insulin (μU/mL) × fasting glucose (mg/dL)/405. Homeostasis model assessment of β-cell function (HOMA-β) (%) was calculated by the previously reported formula: 360 × fasting insulin (μU/mL)/(fasting glucose (mg/dL) – 63). Enzyme-linked immunosorbent assay kits were used for measurement of FABP4 (BioVendor, Czech Republic) and FABP5 (BioVendor). The reproducibility, accuracy and precision of the kits have been reported previously (13, 33).

### Statistical Analysis

Numeric variables were expressed as means with standard deviation (SD) or medians with interquartile ranges. Categorical values were expressed as numbers with percentages, and comparisons of the two group were analyzed by Fisher's exact test or the chi-squared test. Comparisons between two paired groups were analyzed by a two-sample *t*-test. Correlations between two continuous variables were analyzed by using Pearson's correlation coefficients. Multivariable linear regression models were constructed to explore independent parameters of levels of FABPs. Age, sex, and treatment group as well as variables with significant correlations determined by Pearson's coefficients were incorporated in the models. The relationships were expressed with unstandardized regression coefficient, standardized regression coefficient (β) and standard error (SE) of regression coefficient. Statistical analyses were performed at an independent data center (Institute for Clinical Effectiveness, Kyoto, Japan) by study statisticians using SAS 9.4 (SAS Institute Inc, NC) and JMP 13.1 (SAS Institute Inc, NC). All *P*-values were two-sided, and *P* < 0.05 was considered statistically significant.

## Results

### Basal Characteristics of the Patients

Characteristics of the recruited patients are shown in Table 1. Prevalences of hypertension, coronary artery disease and stroke were 77, 44, and 15%, respectively. As medications for dyslipidemia, 80 and 9% of the patients had been treated with a strong statin and ezetimibe, respectively. The use of a DPP-4 inhibitor and an angiotensin II receptor blocker were being administrated in 81 and 51% of the patients, respectively. The female patients were older than the male patients (Table 1). Frequencies of smoking habit, alcohol drinking habit, coronary artery disease and use of a mineralocorticoid receptor antagonist were significantly higher in males than in females. Male patients had significantly higher levels of creatinine and fasting glucose and lower levels of total cholesterol, LDL cholesterol, HDL cholesterol, non-HDL cholesterol, HOMA-β and FABP4 (18.2 [13.2–23.0] vs. 24.6 [19.2–34.7] ng/mL, *P* < 0.01) than did female patients (Supplementary Table 1). No significant difference in FABP5 level was found between male and female patients (6.7 [4.9–8.6] vs. 6.7 [4.7–9.9] ng/mL, *P* = 0.54).

**Table 1:** Background of the patients with type 2 diabetes mellitus (*n* = 256).

	**Total**	**Male**	**Female**	***P***
	**(*n* = 256)**	**(*n* = 146)**	**(*n* = 110)**	
Age (years)	68 ± 10	66 ± 10	70 ± 9	<0.01
Body mass index	26.0 ± 3.7	26.1 ± 3.8	25.9 ± 3.6	0.65
Waist circumference (cm)	92.8 ± 10.1	92.5 ± 10.1	93.3 ± 10.0	0.53
Systolic blood pressure (mmHg)	134 ± 16	133 ± 17	134 ± 15	0.67
Diastolic blood pressure (mmHg)	73 ± 11	74 ± 12	71 ± 10	0.04
Smoking habit	134 (52)	107 (73)	27 (25)	<0.01
Alcohol drinking habit	103 (40)	84 (58)	19 (17)	<0.01
**Diagnosis**
Hypertension	197 (77)	111 (76)	86 (78)	0.69
Dyslipidemia	256 (100)	146 (100)	110 (100)	–
Coronary artery disease	113 (44)	79 (54)	34 (31)	<0.01
Stroke	39 (15)	22 (15)	17 (15)	0.93
**Medication**				
Dipeptidyl peptidase-4 inhibitor	207 (81)	116 (79)	91 (83)	0.51
Biguanide	114 (45)	61 (42)	53 (48)	0.31
Thiazolidinedione	0 (0)	0 (0)	0 (0)	–
α glucosidase inhibitor	35 (14)	23 (16)	12 (11)	0.26
Sulfonylurea	52 (20)	30 (21)	22 (20)	0.91
Glinide	9 (4)	4 (3)	5 (5)	0.50
Sodium-glucose cotransporter 2 inhibitor	30 (12)	20 (14)	10 (9)	0.26
Insulin	22 (9)	15 (10)	7 (6)	0.27
Statin	256 (100)	146 (100)	110 (100)	–
Strong statin^a^	206 (80)	116 (79)	90 (82)	0.64
Ezetimibe	23 (9)	15 (10)	8 (7)	0.41
Fibrate	13 (5)	7 (5)	6 (5)	0.81
Eicosatetraenoic acid	26 (10)	18 (12)	8 (7)	0.18
Angiotensin II receptor blocker	131 (51)	76 (52)	55 (50)	0.74
Angiotensin-converting enzyme inhibitor	25 (10)	17 (12)	8 (7)	0.24
Calcium channel blocker	119 (46)	64 (44)	55 (50)	0.33
β blocker	65 (25)	41 (28)	24 (22)	0.25
Diuretic	40 (16)	25 (17)	15 (14)	0.45
Mineralocorticoid receptor antagonist	11 (4)	10 (7)	1 (0.9)	0.03

*Variables are expressed as number (%), means ± SD, or medians (interquartile ranges)*.

a *Indicates atorvastatin, rosuvastatin, and pitavastatin*.

### Correlation and Multivariable Linear Regression Analyses for FABP4 Level

**Figure 1:**
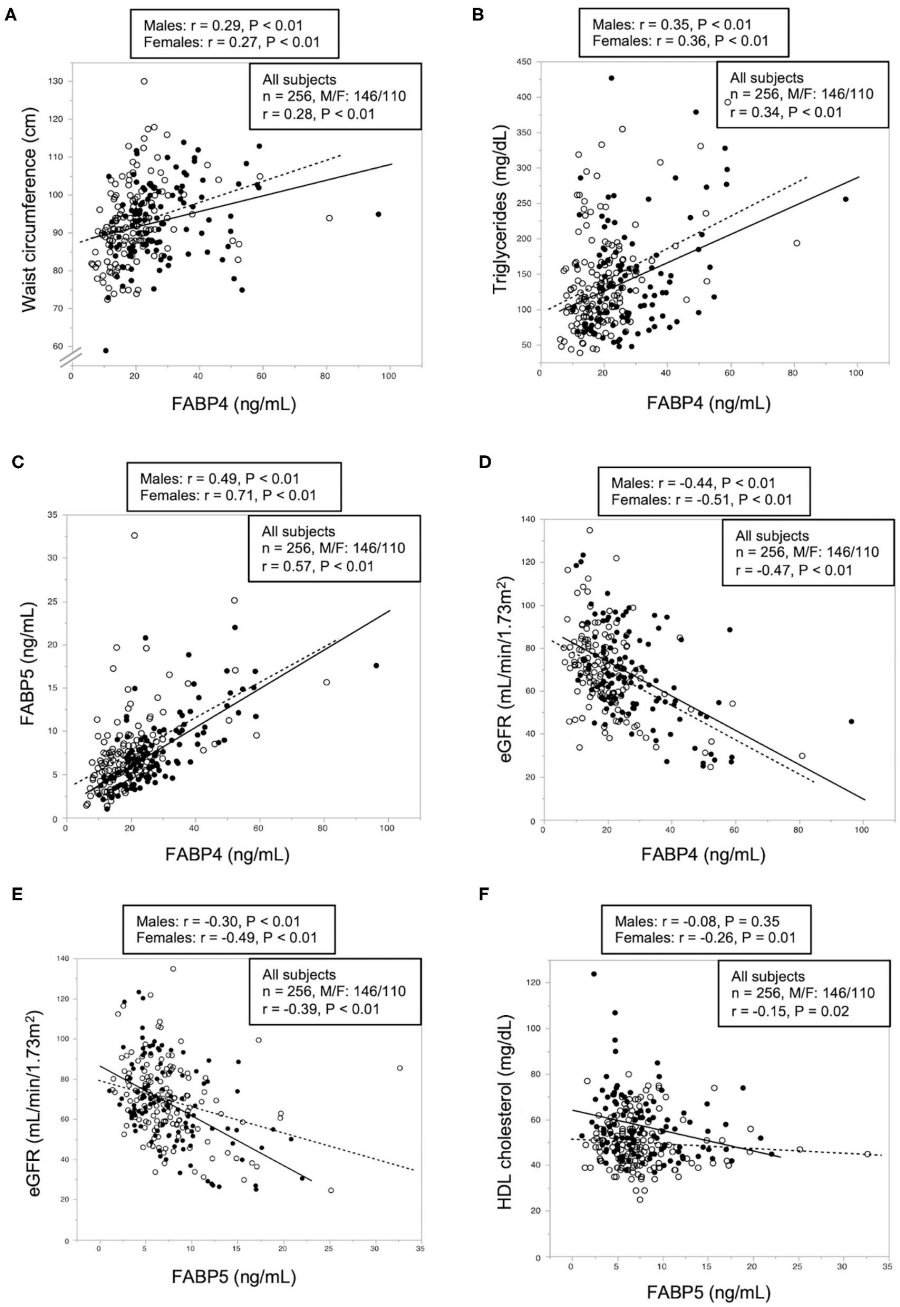
Correlations of FABP4 and FABP5 levels with metabolic parameters. **(A–D)** Waist circumference **(A)** and levels of triglycerides **(B)**, fatty acid-binding protein 5 (FABP5) **(C)** and estimated glomerular filtration rate (eGFR) **(D)** were plotted against fatty acid-binding protein 4 (FABP4) level in each subject (*n* = 256). **(E,F)** Levels of eGFR **(E)** and high-density lipoprotein (HDL) cholesterol level **(F)** were plotted against FABP5 level in each subject (*n* = 256). Open circles and broken regression line: males (*n* = 146), closed circles and solid regression line: females (*n* = 110).

As shown in Table 2, level of FABP4 was positively correlated with age, BMI, waist circumference (Figure 1A) and levels of blood urea nitrogen, creatinine, total cholesterol, LDL cholesterol, triglycerides (Figure 1B), non-HDL cholesterol and FABP5 (Figure 1C) and was negatively correlated with eGFR (Figure 1D) in all of the patients. No significant correlation of FABP4 level with blood pressures, AST, ALT, γGTP, fasting glucose, insulin, HOMA-R, HOMA-β, or HbA1c was found (Table 2). Similar correlations between the parameters except diastolic blood pressure, total cholesterol, LDL cholesterol, HDL cholesterol and non-HDL cholesterol were found when sex was separately analyzed.

**Table 2:** Correlation analysis for FABP4.

	**Total (*****n*** **=** **256)**		**Male (*****n*** **=** **146)**		**Female (*****n*** **=** **110)**	
	***r***	***P***	***r***	***P***	***r***	***P***
Age	0.18	<0.01	0.02	0.79	0.22	0.02
Body mass index	0.22	<0.01	0.17	0.04	0.34	<0.01
Waist circumference	0.28	<0.01	0.29	<0.01	0.27	<0.01
Systolic blood pressure	−0.10	0.11	−0.08	0.32	−0.16	0.10
Diastolic blood pressure	−0.12	0.06	0.02	0.80	−0.21	0.02
AST	0.09	0.16	0.07	0.43	0.10	0.29
ALT	−0.01	0.86	−0.01	0.92	0.05	0.63
γGTP	0.08	0.19	0.13	0.12	0.13	0.19
Blood urea nitrogen	0.41	<0.01	0.49	<0.01	0.40	<0.01
Creatinine	0.39	<0.01	0.59	<0.01	0.57	<0.01
eGFR	−0.47	<0.01	−0.44	<0.01	−0.51	<0.01
Total cholesterol	0.24	<0.01	0.31	<0.01	0.00	0.97
LDL cholesterol	0.15	0.02	0.18	0.03	−0.01	0.90
HDL cholesterol	−0.01	0.91	0.08	0.33	−0.26	0.01
Triglycerides	0.34	<0.01	0.35	<0.01	0.36	<0.01
Non-HDL cholesterol	0.28	<0.01	0.31	<0.01	0.15	0.11
Fasting glucose	−0.05	0.46	−0.10	0.22	0.10	0.32
Insulin	−0.01	0.87	−0.04	0.63	0.14	0.14
HOMA-R	−0.03	0.58	−0.05	0.53	0.09	0.36
HOMA-β	0.10	0.10	0.01	0.95	0.12	0.22
Hemoglobin A1c	0.03	0.60	−0.02	0.85	0.06	0.54
FABP5	0.57	<0.01	0.49	<0.01	0.71	<0.01

*AST, aspartate transaminase; ALT, alanine transaminase; eGFR, estimated glomerular filtration rate; FABP, fatty acid-binding protein; γGTP, γ-glutamyl transpeptidase; HDL, high-density lipoprotein; HOMA-β, homeostasis model assessment of β-cell function; HOMA-R, homeostasis model assessment of insulin resistance; LDL, low-density lipoprotein*.

Multivariable linear regression analysis using age, sex, waist circumference, eGFR, total cholesterol, triglycerides, and FABP5 demonstrated that waist circumference (β = 0.21, *P* < 0.01) and levels of eGFR (β = −0.31, *P* < 0.01), triglycerides (β = 0.16, *P* < 0.01), and FABP5 (β = 0.39, *P* < 0.01) were independent predictors of FABP4 level after adjustment of sex and age (*R*^2^ = 0.579) (Table 3).

**Table 3:** Multivariable regression analysis for FABP4.

	**Regression coefficient**	**SE**	**Standardized regression coefficient (β)**	***P***
Age	−0.06	0.06	−0.05	0.32
Sex (Male)	−7.04	1.13	−0.28	<0.01
Waist circumference	0.26	0.05	0.21	<0.01
eGFR	−0.19	0.03	−0.31	<0.01
Total cholesterol	0.02	0.02	0.04	0.45
Triglycerides	0.03	0.01	0.16	<0.01
FABP5	1.16	0.14	0.39	<0.01

*R^2^ = 0.579*.

*eGFR, estimated glomerular filtration rate; FABP, fatty acid-binding protein*.

### Correlation and Multivariable Linear Regression Analyses for FABP5 Level

As shown in Table 4, FABP5 concentration was positively correlated with age, waist circumference and levels of blood urea nitrogen, creatinine, triglycerides, non-HDL cholesterol and FABP4 and was negatively correlated with diastolic blood pressure and levels of eGFR (Figure 1E) and HDL cholesterol (Figure 1F) in all of the patients. No significant correlation of FABP5 level with BMI, systolic blood pressure, ALT, AST, γGTP, total cholesterol, LDL cholesterol, fasting glucose, insulin, HOMA-R, HOMA-β, or HbA1c was found (Table 4). When divided by sex, FABP5 level was positively correlated with age, waist circumference, BMI and HbA1c, and was negatively correlated with HDL cholesterol in female patients but not in male patients.

**Table 4:** Correlation analysis for FABP5.

	**Total (*****n*** **=** **256)**		**Male (*****n*** **=** **146)**		**Female (*****n*** **=** **110)**	
	***r***	***P***	***r***	***P***	***r***	***P***
Age	0.17	0.01	0.08	0.31	0.29	<0.01
Body mass index	0.01	0.86	−0.14	0.10	0.23	0.02
Waist circumference	0.12	<0.05	0.04	0.65	0.23	0.02
Systolic blood pressure	−0.09	0.14	−0.17	0.04	0.02	0.83
Diastolic blood pressure	−0.15	0.02	−0.15	0.06	−0.13	0.19
AST	0.04	0.54	0.00	0.97	0.10	0.32
ALT	−0.04	0.49	−0.07	0.40	0.00	0.97
γGTP	0.03	0.59	0.00	0.98	0.08	0.39
Blood urea nitrogen	0.41	<0.01	0.36	<0.01	0.49	<0.01
Creatinine	0.42	<0.01	0.40	<0.01	0.54	<0.01
eGFR	−0.39	<0.01	−0.30	<0.01	−0.49	<0.01
Total cholesterol	0.09	0.15	0.18	0.03	−0.04	0.64
LDL cholesterol	0.09	0.17	0.17	0.04	−0.04	0.69
HDL cholesterol	−0.15	0.02	−0.08	0.35	−0.26	0.01
Triglycerides	0.23	<0.01	0.18	0.03	0.30	<0.01
Non-HDL cholesterol	0.18	<0.01	0.23	<0.01	0.10	0.28
Fasting glucose	0.00	0.96	−0.08	0.33	0.13	0.19
Insulin	0.03	0.66	0.01	0.91	0.12	0.20
HOMA-R	0.00	0.97	−0.01	0.90	0.08	0.42
HOMA-β	0.05	0.40	0.06	0.45	0.04	0.66
Hemoglobin A1c	0.07	0.28	−0.05	0.56	0.23	0.01
FABP4	0.57	<0.01	0.49	<0.01	0.71	<0.01

*AST, aspartate transaminase; ALT, alanine transaminase; eGFR, estimated glomerular filtration rate; FABP, fatty acid-binding protein; γGTP, γ-glutamyl transpeptidase; HDL, high-density lipoprotein; HOMA-β, homeostasis model assessment of β-cell function; HOMA-R, homeostasis model assessment of insulin resistance; LDL, low-density lipoprotein*.

Multivariable linear regression analysis using age, sex, waist circumference, diastolic blood pressure, eGFR, HDL cholesterol, triglycerides, and FABP4 demonstrated that levels of HDL cholesterol (β = −0.12, *P* = 0.03) and FABP4 (β = 0.57, *P* < 0.01) were independent predictors of FABP5 level after adjustment of sex and age (*R*^2^ = 0.397) (Table 5).

**Table 5:** Multivariable regression analysis for FABP5.

	**Regression coefficient**	**SE**	**Standardized regression coefficient (β)**	***P***
Age	0.01	0.03	0.02	0.73
Sex (Male)	1.05	0.49	0.12	0.03
Waist circumference	−0.01	0.02	−0.03	0.62
Diastolic blood pressure	−0.02	0.02	−0.06	0.28
eGFR	−0.02	0.01	−0.11	0.10
HDL cholesterol	−0.04	0.02	−0.12	0.03
Triglycerides	0.001	0.003	0.02	0.75
FABP4	0.19	0.02	0.57	<0.01

*R^2^ = 0.397*.

*eGFR, estimated glomerular filtration rate; FABP, fatty acid-binding protein; HDL, high-density lipoprotein*.

## Discussion

The present study demonstrated that concentrations of FABP4 and FABP5 were independent predictors of each other. Furthermore, distinct independent associations of FABP4 concentration with adiposity, renal dysfunction and hypertriglyceridemia and a distinct independent association of FABP5 level with a low HDL level were found in type 2 diabetic patients at high risks for cardiovascular disease who were receiving statin therapy. The similarity of amino acids between FABP4 and FABP5 is 52%, and both proteins bind to several fatty acids with similar affinity and selectivity (1). FABP4 and FABP5 are expressed in adipocytes and macrophages, though the amounts of these proteins are different (4, 5, 40, 41). The stoichiometry of FABP4 is about 100-fold larger than that of FABP5 in adipocytes (40). The amount of FABP4 and that of FABP5 are almost same in macrophages under usual conditions (5), though the amount of FABP4 in macrophages is about 10,000-fold smaller than that in adipocytes (41). In addition, deficiency of FABP4 causes a compensatory induction of FABP5 in adipocytes (4). Taken together, the results showing that circulating levels of FABP4 and FABP5 were independent predictors of each other are reasonable. Previous studies also revealed a positive correlation between concentrations of FABP4 and FABP5 in several populations (32–34, 42, 43).

The results showing distinct independent associations of increased FABP4 level with female gender, waist circumference as an index of adiposity, renal dysfunction indicated by low eGFR, and hypertriglyceridemia were in agreement with results of previous studies showing the same associations in a general population and in patients with several metabolic disorders (13, 14, 22, 23), though the correlations were not strong probably due to the modulation of several concomitant drugs for diabetes mellitus, dyslipidemia and hypertension in the present study as a real-world setting. It has been shown that circulating FABP5 exists at levels of about a half or less of circulating FABP4 (14, 32–34, 42, 43), as was confirmed in the present study. It has been shown that FABP5 concentration are related to several metabolic markers, though the correlation is not stronger than that of FABP4 (14, 33, 34). Interestingly, the concentration of FABP5, but not that of FABP4, has been reported to be negatively and independently associated with cholesterol efflux capacity from macrophages as a function of HDL cholesterol, suggesting a potential residual risk biomarker in atherosclerosis (32). In the present study, the FABP5 concentration, but FABP4 concentration, was independently and negatively associated with HDL cholesterol level, though cholesterol efflux capacity was not investigated.

Dual ablation of FABP4 and FABP5 was shown to protect from metabolic disorders including insulin resistance, fatty liver, and atherosclerosis more than does a single deficiency of FABP4 or that of FABP5 in mouse models (4, 5, 44–46). Local actions of FABP4 and FABP5 in macrophages and those in adipocytes distinctly integrate metabolic and inflammatory responses to regulate insulin sensitivity (47). It has recently been suggested that manipulation of FABP4 by using neutralizing antibodies, specific inhibitors or unidentified receptor blockers would be a novel therapeutic strategy for several cardiovascular and metabolic diseases (3). A further investigation of the mechanism of integrated actions of FABP4 and FABP5 may enable the development of new therapeutic strategies for metabolic disease and atherosclerotic cardiovascular disease.

There are some limitations in the present study. First, since this is a cross-sectional study, causal association between concentrations of FABP4 and FABP5 and the correlated biomarkers could not be proved. Longitudinal and interventional studies are necessary to clarify the relationships of FABP4 and FABP5 with metabolic parameters. Second, patients who had been treated with a thiazolidinedione, which is a PPARγ agonist, were excluded in the present study since FABP4 is a target of PPARγ (37, 38). However, most of the recruited patients had been treated with therapeutic drugs including antidiabetic drugs (24–27), statins (28), omega-3 fatty acids (12), and angiotensin II receptor blockers (30), which have been shown to affect FABP4 level. Therefore, those drugs might have affected levels and correlations of FABPs.

In conclusion, circulating FABP4 and FABP5 levels are independent predictors each other in patients with type 2 diabetes mellitus. There are distinct independent associations of FABP4 level with renal dysfunction, adiposity and high triglycerides level and there is a distinct association of FABP5 level with a low HDL level. A further investigation of the mechanism underlying the link between levels of FABP4 and FABP5 and metabolic parameters may lead to the development of new therapeutic strategies for metabolic and atherosclerotic cardiovascular diseases.

## Data Availability Statement

The datasets analyzed during the current study are available from the corresponding author on reasonable request (furuhasi@sapmed.ac.jp).

## Ethics Statement

The studies involving human participants were reviewed and approved by the institutional review boards at the University of the Ryukyus (No. 731) and each participating center. The patients/participants provided their written informed consent to participate in this study.

## Author Contributions

TM and MSa carried out the statistical analyses. MF prepared the first draft of the manuscript. All authors made substantial contributions to the conception, design, and acquisition and interpretation of data, participated in reviewing the manuscript and take full responsibility for its content, read, and approved the final manuscript.

## Conflict of Interest

The REASON trial was funded by Kowa Company, Ltd, which had no role in study design, data collection and analysis, decision to publish, or preparation of the manuscript in the present study. MF reports non-purpose research grants from Astellas, Mitsubishi Tanabe, Sanwa Kagaku Kenkyusho, and MediciNova; lecturer's fees from Mitsubishi Tanabe, Kowa, Mochida, Daiichi Sankyo, Novartis, Boehringer Ingelheim, MSD, Sanwa Kagaku Kenkyusho, Takeda, Astellas, Sanofi, and AstraZeneca. IS reports research grants from Public Health Research Foundation, Kowa, National Cerebral and Cardiovascular Center and Medical Informatics Study Group; non-purpose research grants from Public Health Research Foundation, Eastep, Nexis, Takeda, Daiichi Sankyo, Beohringer Ingelheim, AstraZeneca, MSD, Amgen, Astellas, Sanofi, Fuji, and Novartis; lecturer's fees from AstraZeneca, Takeda, Bayer, Pfizer, Bristol-Myers Squibb, Boehringer lngelheim, MSD, Kyowa Hakko Kirin, Daiichi Sankyo, Novartis, Sanofi, Kowa, Shionogi, Kissei, Astellas, Amgen, Ono, Otsuka, Novonordisk, Mochida, Teijin, Sysmex, Nipro, Kyorin, Fuji, and Sumitomo Dainippon; advisory board for Public Health Research Foundation, Kowa, Tanabe, Kyowa Hakko Kirin, and Bristol-Myers Squibb, Sysmex. TM reports lecturer's fees from Bayer, Daiichi Sankyo, Japan Lifeline, Kyocera, Mitsubishi Tanabe, Novartis, and Toray; manuscript fees from Bristol-Myers Squibb and Kowa; advisory boards for Asahi Kasei, Boston Scientific, Sanofi, and Bristol-Myers Squibb. MSh reports research grants from AstraZeneca, Ono, and Sanwa Kagaku Kenkyusho; non-purpose research grants from Astellas, AstraZeneca, Bayer, Boehringer Ingelheim, Chugai, Eli Lilly, Kowa, Mitsubishi Tanabe, MSD, Novo Nordisk, Ono, Taisho Toyama, and Takeda; lecturer's fees from Astellas, AstraZeneca, Bayer, Boehringer Ingelheim, Chugai, Eli Lilly, Kowa, Mitsubishi Tanabe, Mochida, MSD, Novo Nordisk, Ono, Taisho Toyama, and Takeda; advisory board for Novo Nordisk; sponsored office from Boehringer Ingelheim. TN reports research grants from Eli Lilly, Mitsubishi Tanabe, MSD, and Novartis; lecturer's fees from Arkray, Astellas, AstraZeneca, Boehringer Ingelheim, Eli Lilly, Johnson & Johnson, Mitsubishi Tanabe, MSD, Novartis, Novo Nordisk, Ono, Sanofi, Sanwa Kagaku Kenkyusho, Sumitomo Dainippon, Taisho Toyama, Takeda, and Terumo. OA reports lecturer's fees from Abbott, Astellas, Boehringer Ingelheim, Medtronic, and St. Jude Medical. KN reports research grants from Actelion, Asahi Kasei, Astellas, Astellas Amgen Bio Pharma, Bayer, Boehringer Ingelheim, GlaxoSmithKline, Mitsubishi Tanabe, Novo Nordisk, Teijin, and Terumo; non-purpose research grants from Astellas, Bayer, Boehringer Ingelheim, Bristol-Myers Squibb, Daiichi Sankyo, Eisai, Eli Lilly, Japan Lifeline, Mitsubishi Tanabe, MSD, Novartis, Novo Nordisk, Ono, Otsuka, Pfizer, Sanofi, Sumitomo Dainippon, Takeda, and Teijin; lecturer's fees from Actelion, Astellas, Astellas Amgen Bio Pharma, AstraZeneca, Bayer, Boehringer Ingelheim, Bristol-Myers Squibb, Daiichi Sankyo, Edwards Lifesciences, Eli Lilly, FUJIFILM, Fukuda Denshi, Kowa, Kyowa Hakko Kirin, Mebix, Medtronic, Mitsubishi Tanabe, Mochida, MSD, Novartis, Novo Nordisk, Ono, Otsuka, Pfizer, Roche Diagnostics, Sanofi, Sanwa Kagaku Kenkyusho, Sumitomo Dainippon, Taisho Toyama, Takeda, and Teijin; manuscript fee from Astellas, and Takeda; advisory board for Astellas, AstraZeneca, Boehringer Ingelheim, Eli Lilly, Mitsubishi Tanabe, MSD, Novo Nordisk, Pfizer, and Takeda. SU reports research grants from Bristol-Myers Squibb, and Kowa; non-purpose research grants from Bristol-Myers Squibb, Chugai, MSD, Pfizer, and Takeda; lecturer's fees from Boehringer Ingelheim, MSD, and Taiho; manuscript fees from Kowa; advisory board for Otsuka. The remaining authors declare that the research was conducted in the absence of any commercial or financial relationships that could be construed as a potential conflict of interest.
