# Supplementary material for: Independent and Distinct Associations of FABP4 and FABP5 With Metabolic Parameters in Type 2 Diabetes Mellitus
Source: Front Endocrinol (Lausanne). 2020 Sep 23;11:575557. doi: 10.3389/fendo.2020.575557 (PMC7538548; doi:10.3389/fendo.2020.575557)
Supplement: Supplementary file 1 [file Data_Sheet_1.PDF]

Supplementary Table 1

Supplementary Table S1. Biochemical data

	Total (n = 256)	Male (n = 146)	Female (n = 110)	*P*
AST (IU/L)	21 (18 - 27)	21 (18 - 27)	21 (18 - 27)	0.64
ALT (IU/L)	20 (15 - 31)	21 (16 - 33)	19 (15 - 27)	0.18
γGTP (IU/L)	26 (18 - 43)	31 (22 - 46)	22 (17 - 34)	0.06
Blood urea nitrogen (mg/dL)	16.6 ± 5.5	16.7 ± 5.7	16.4 ± 5.4	0.71
Creatinine (mg/dL)	0.83 ± 0.27	0.91 ± 0.25	0.72 ± 0.25	< 0.01
eGFR (mL/min/1.73m^2)	68.6 ± 19.7	69.6 ± 18.6	67.4 ± 21.1	0.38
Total cholesterol (mg/dL)	189 ± 29	181 ± 28	198 ± 28	< 0.01
LDL cholesterol (mg/dL)	112 ± 21	108 ± 21	117 ± 22	< 0.01
HDL cholesterol (mg/dL)	53 ± 13	50 ± 11	57 ± 14	< 0.01
Triglycerides (mg/dL)	127 (90 - 169)	127 (96 - 167)	128 (88 - 177)	0.90
non-HDL cholesterol (mg/dL)	136 ± 25	131 ± 25	141 ± 24	< 0.01
Fasting glucose (mg/dL)	140 ± 41	145 ± 40	134 ± 42	0.03
Insulin (μU/mL)	7.6 (5.0 - 11.9)	7.4 (4.6 - 12.3)	8.1 (5.3 - 11.1)	0.53
HOMA-R	2.52 (1.54 - 4.09)	2.51 (1.53 - 4.53)	2.53 (1.58 - 3.76)	0.84
HOMA-β (%)	39.9 (26.2 - 67.8)	38.3 (22.6 - 65.6)	44.9 (29.7 - 83.3)	0.03
Hemoglobin A1c (%)	7.0 ± 0.8	6.9 ± 0.8	7.0 ± 0.8	0.48
FABP4 (ng/mL)	20.4 (14.9 - 26.7)	18.2 (13.2 - 23.0)	24.6 (19.2 - 34.7)	< 0.01
FABP5 (ng/mL)	6.7 (4.8 - 9.1)	6.7 (4.9 - 8.6)	6.7 (4.7 - 9.9)	0.54

Variables are expressed as means ± SD or medians (interquartile ranges).
AST, aspartate transaminase; ALT, alanine transaminase; eGFR, estimated glomerular filtration rate; FABP, fatty acid-binding protein; γGTP, γ-glutamyl transpeptidase; HDL, high-density lipoprotein; HOMA-β, homeostasis model assessment of β-cell function; HOMA-R, homeostasis model assessment of insulin resistance; LDL, low-density lipoprotein.
